# Introduction to Special Issue: 2016 Translational Medicine and Bioengineering Conference

**DOI:** 10.1002/btm2.10071

**Published:** 2017-07-25

**Authors:** Kaushal Rege, Samir Mitragotri

**Affiliations:** ^1^ Chemical Engineering, Arizona State University, Tempe, AZ 85287; ^2^ John A Paulson School of Engineering & Applied Sciences, Wyss Institute, Harvard University Cambridge, MA 02138

*Bioengineering & Translational Medicine* was launched in 2016 with the goal to highlight the scientific and technological issues relevant to the translational challenges of bioengineering science and technologies. Clinical translation of promising biological technologies can have a transformative impact on human health. Several advances in bioengineering have already had a huge impact in the diagnosis and treatment of human diseases. However, with rapid development of new technologies coming down the innovation pipeline, it is critical to develop streamlined pathways for their eventual translation to the clinic. The journal has been met with significant excitement by the scientific community.

Last year saw another milestone for AIChE and SBE in the field of *Bioengineering and Translational Medicine*. Specifically, a new scientific meeting, Translational Medicine and Bioengineering Conference, was launched to further highlight the significance of this topic. The conference was organized on November 12 and 13, 2016 in San Francisco, CA (https://www.aiche.org/sbe/conferences/translational-medicine-and-bioengineering-conference/2016), just ahead of AIChE's annual meeting. It brought together scientists, bioengineers, leaders from industry, and various stakeholders as part of a single‐track conference format at the interface of innovative biological technologies and clinical translation.

Professors Ravi Kane and Mark Prausnitz from Georgia Tech were the inaugural co‐chairs of the conference, and along with individual session co‐chairs, organized an exciting program that brought forth cutting‐edge translational bioengineering technologies in biopharmaceuticals, gene and drug delivery, immunoengineering, biomanufacturing, and stem cells and regenerative medicine in technical sessions dedicated to each of these topics. Each session had a strong mix of invited speakers as well as contributed platform presentations. The program also included a panel discussion on entrepreneurship in which entrepreneurs from academia, industrial leaders, and technology transfer specialists shared invaluable thoughts and experiences in the path traveled from the lab bench to the clinic. The conference provided a unique platform for bioengineers to present innovative advances at different stages of the translational spectrum.

This special issue of *BioTM* features contributions from some of the participants in this conference as well as other scientists in the field. Prof. Christopher Jewell (U. Maryland) and coworkers review the latest advances in immunomodulatory biomaterials for applications in regenerative medicine and tissue engineering.^1^ Prof. Subbu Venkatraman (Nanyang Technological University, Singapore) and coworkers' timely review on translation of stents and occluders covers key advances of these devices in in cardiovascular medicine.^2^ In addition to these outstanding review articles, this special issue also includes six research reports. Prof. Eniola‐Adefeso (U. Michigan) and coworkers report a detailed molecular‐level study of adhesion of PLGA nanoparticles in human blood flow, with significant implications for drug delivery.^3^ Prof. Robert E. Atkins Jr. (U. Delaware) and coworkers describe the use of a hydrogel for modulating the elasticity or arteries following isolation using skeletonization.^4^ Prof. Jacob Elmer (U. Villanova) and coworkers compare different extracellular oxygen carriers from invertebrates as potential blood substitutes.^5^ Prof. Steven Jay (U. Maryland) and coworkers report the impact of biomanufacturing‐related operating parameters on the production and biological activity of extracellular vesicles derived from mesenchymal stem cells.^6^ Prof. Sean Palecek (U. Wisconsin) and coworkers describe the generation of endothelial cells from human pluripotent stem cell‐derived epicardial cells.^7^ Prof. Benjamin Keselowsky (U. Florida) and coworkers report the development of blend polymeric microparticles for T‐cell activation.^8^

We invite you to read the excellent array of review and research articles in this issue of *Bioengineering and Translational Medicine*.

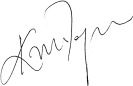

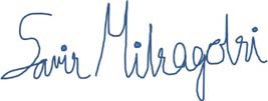

## References

[1] Andorko JI , Jewell CM. Designing biomaterials with immunomodulatory properties for tissue engineering and regenerative medicine. Bioeng Transl Med. 2017;2(2):139–155. doi: 10.1002/btm2.10063 28932817 10.1002/btm2.10063 PMC5579731

[2] Huang Y , Wong YS , Ng HCA , Boey FYC , Venkatraman S. Translation in cardiovascular stents and occluders: From biostable to fully‐degradable. Bioeng Transl Med. 2017;2(2):156–169. doi: 10.1002/btm2.10066 10.1002/btm2.10066 PMC5675095 29313029

[3] Sobczynski DJ , Eniola‐Adefeso O. IgA and IgM protein primarily drive plasma corona‐induced adhesion reduction of PLGA nanoparticles in human blood flow. Bioeng Transl Med. 2017;2(2):180–190. doi: 10.1002/btm2.10064 28932819 10.1002/btm2.10064 PMC5579729

[4] Robinson KG , Scott RA , Hesek AM , et al. Reduced arterial elasticity due to surgical skeletonization is ameliorated by abluminal PEG hydrogel. Bioeng Transl Med. 2017;2(2):222–232. doi: 10.1002/btm2.10060 28932820 10.1002/btm2.10060 PMC5579730

[5] Zimmerman D , DiIusto M , Dienes J , Abdulmalik O , Elmer JJ. Direct comparison of oligochaete erythrocruorins as potential blood substitutes. Bioeng Transl Med. 2017;2(2):212–221. doi: 10.1002/btm2.10067 10.1002/btm2.10067 PMC5675092 29313031

[6] Patel DB , Gray KM , Santharam Y , Lamichhane TN , Stroka KM , Jay SM. Impact of cell culture parameters on production and vascularization bioactivity of mesenchymal stem cell‐derived extracellular vesicles. Bioeng Transl Med. 2017;2(2):170–179. doi: 10.1002/btm2.10065 28932818 10.1002/btm2.10065 PMC5579732

[7] Bao X , Bhute VJ , Han T , Qian T , Lian X , Palecek SP. Human pluripotent stem cell‐derived epicardial progenitors can differentiate to endocardial‐like endothelial cells. Bioeng Transl Med. 2017;2(2):191–201. doi: 10.1002/btm2.10062 10.1002/btm2.10062 PMC5675097 29170757

[8] Yang L , Bracho‐Sanchez E , Fernando LP , et al. Poly(2‐propylacrylic acid)/poly(lactic‐co‐glycolic acid) blend microparticles as a targeted antigen delivery system to direct either CD4+ or CD8+ T cell activation. Bioeng Transl Med. 2017;2(2):202–211. doi: 10.1002/btm2.10068 10.1002/btm2.10068 PMC5675098 29313030

